# Aerosol effect on the evolution of the thermodynamic properties of warm convective cloud fields

**DOI:** 10.1038/srep38769

**Published:** 2016-12-08

**Authors:** Guy Dagan, Ilan Koren, Orit Altaratz, Reuven H. Heiblum

**Affiliations:** 1 Department of Earth and Planetary Sciences, The Weizmann Institute of Science, Rehovot 76100, Israel

## Abstract

Convective cloud formation and evolution strongly depend on environmental temperature and humidity profiles. The forming clouds change the profiles that created them by redistributing heat and moisture. Here we show that the evolution of the field’s thermodynamic properties depends heavily on the concentration of aerosol, liquid or solid particles suspended in the atmosphere. Under polluted conditions, rain formation is suppressed and the non-precipitating clouds act to warm the lower part of the cloudy layer (where there is net condensation) and cool and moisten the upper part of the cloudy layer (where there is net evaporation), thereby destabilizing the layer. Under clean conditions, precipitation causes net warming of the cloudy layer and net cooling of the sub-cloud layer (driven by rain evaporation), which together act to stabilize the atmosphere with time. Previous studies have examined different aspects of the effects of clouds on their environment. Here, we offer a complete analysis of the cloudy atmosphere, spanning the aerosol effect from instability-consumption to enhancement, below, inside and above warm clouds, showing the temporal evolution of the effects. We propose a direct measure for the magnitude and sign of the aerosol effect on thermodynamic instability.

A warm convective cloud forms when a rising parcel with humid air cools and reaches saturation. The likelihood of such a parcel rising, and its properties above the cloud base, depend on the perturbation that pushed the parcel upward and on the instability of the atmospheric thermodynamic profile (often expressed by the temperature lapse rate or convective available potential energy - CAPE - which measures the total potential buoyant energy of an environment^1^). However, another important component controlling the cloud’s properties is linked to the system’s microphysical properties. The efficiency of the transfer of water vapor molecules to a liquid drop and therefore, the flux of latent heat release (which further fuels the parcel’s buoyancy) depend on the suspended aerosol properties^2^,^3^,^4^,^5^. Aerosols serve as cloud condensation nuclei (CCN), as they reduce the supersaturation required for cloud droplet formation (droplet activation). Without CCN, an air parcel would require supersaturation levels of a few hundred percent to allow for formation of stable droplets by spontaneous sticking of water molecules^6^. Moreover, the aerosol concentration, size distribution and composition control the consequent cloud drop concentration and size distribution and hence the drops’ terminal velocity distribution. This will dictate the mobility of the cloud’s liquid water, and in particular how fast the liquid water is lifted by the air’s updraft during the cloud’s growing stages^7^. Furthermore, aerosol regulates the timing and likelihood of a significant occurrence of the stochastic collision–coalescence events required for rain formation^8^,^9^,^10^,^11^,^12^,^13^.

The focus here is on warm convective clouds. These clouds are frequent over the oceans^14^ and play an important role in the lower atmosphere energy and moisture budgets. In addition, they are responsible for the largest uncertainty in tropical cloud feedbacks in climate models^15^.

The interplay between aerosol effects and thermodynamic control in warm convective cloud fields can be separated into two characteristic scales: 1) the coupling between microphysics and dynamics on a single-cloud scale and 2) how the outcomes of such coupling propagate to the cloud-field scale and as a result, how the field’s thermodynamic properties evolve with time. Moreover, on the cloud-field scale, there is an additional source of complexity as the overall cloud-field properties depend not only on the average thermodynamic properties but also on their spatial distribution. Self-organization (i.e. aggregation of clouds or organization in special shapes as arcs) of convective cells can determine the location, size and number of clouds in the field^16^.

On a single warm-cloud scale, the net aerosol effect has been recently shown to have an optimal aerosol concentration (*N*_*op*_) at which clouds reach their maximum development (measured by total liquid mass, updrafts, size or rain yield) per given thermodynamic conditions^5^,^17^. A non-monotonic trend in clouds’ response to changes in aerosol loading was shown for deep clouds as well^18^,^19^,^20^. Warm clouds forming under aerosol concentration values lower than *N*_*op*_ can be viewed as aerosol-limited^2^, whereas for concentrations above *N*_*op*_, the enhanced water loading^5^ and the aerosol-driven enhancement in mixing with non-cloudy, drier air, suppresses cloud development^21^,^22^,^23^. *N*_*op*_ is a function of the thermodynamic conditions, such that conditions that support larger clouds also dictate larger *N*_*op*_ values.

Clouds affect the thermodynamic conditions of the environment in which they reside^24^,^25^,^26^,^27^,^28^,^29^,^30^. The effects evolve with time, and each generation of clouds changes the environmental conditions encountered by the next generation of clouds. Previous studies have shown a “preconditioning” or “cloud deepening”^29^,^31^,^32^,^33^ effect which refers to moistening of the upper part of the cloudy layer and raising the inversion base height. The magnitude of such effects depends on the clouds’ microphysical properties^34^,^35^,^36^. Along the same lines, several studies have made use of observational data and numerical simulations of cloud fields to investigate the role of warm convective clouds in moistening the free troposphere^37^,^38^,^39^,^40^,^41^,^42^, and feedbacks between clouds and the environmental conditions for deep convective clouds^43^,^44^,^45^.

Clouds impacts on their environment are also clearly evident below their bases, as evaporative cooling of rain^28^ can produce cold pools near the surface that can change the organization of the field^16^,^46^. On the one hand, convective thermals originating within these cold pools have a reduced likelihood of reaching the lifting condensation level (LCL) and forming new clouds. On the other, generation of clouds at the cold pool’s boundaries may be enhanced^16^,^47^.

As has been shown theoretically, even low rain rates can significantly affect the thermodynamic structure of trade-wind boundary-layer profiles^48^. Under conditions of precipitation, due to the latent heat release and water removal, the cloudy layer becomes warmer, drier, and more stable compared to non-precipitation conditions. It has also been shown that under non-precipitation conditions, the inversion base height increases due to increased evaporation and cooling above it^48^.

As aerosols change the clouds’ development and rain properties, they are also likely to affect the clouds’ interactions with their thermodynamic environment^34^,^35^. This issue is examined in this work.

The overall aerosol effect on clouds and in particular, its synergy with the environmental thermodynamic conditions, poses one of the largest challenges in our understanding of climate^49^. On the one hand, we are facing climate change and therefore temperature and humidity profiles are changing; on the other, global industry is changing and therefore, so are global aerosol distribution, loadings, and properties. In this work, using a large eddy simulation (LES) model with a detailed bin-microphysics scheme (see details in Methodology), we explore the coupled microphysical–dynamic system on a cloud-field scale and its sensitivity to aerosol loading. We focus on the evolution of temperature and humidity profiles which determine much of the environmental thermodynamic properties, and study how changes driven by the coupling of microphysics and dynamics on smaller scales propagate and affect the way in which clouds change their environment.

## Results and Discussion

For the sake of clarity, the analysis of aerosol effects on the evolution of the thermodynamic profiles (through their impact on clouds) is separated into three different layers: (1) below the cloud base (sub-cloud layer), (2) the main part of the cloudy layer and (3) cloud top (upper cloudy layer) and the inversion layer.

**Figure 1:**
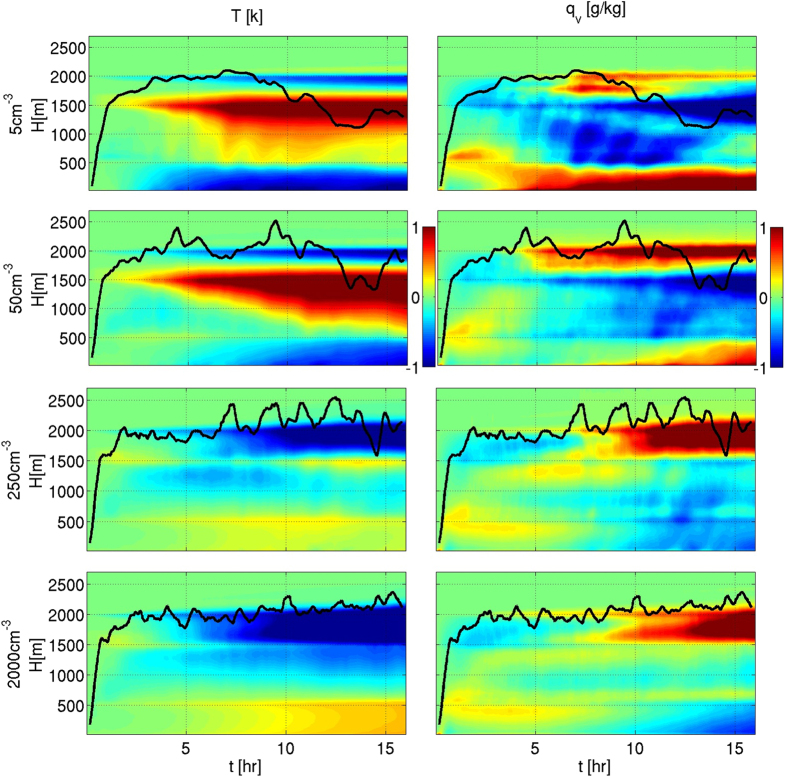
Temporal changes compared to the initial profiles of mean environmental temperature [K] (left column) and mean water vapor mixing ratio [g/kg] (right column). Each row shows the temporal evolution of the differences for a given aerosol concentration (5, 50, 250 and 2000 cm^−3^). Black lines present the 10 minutes running average of the maximum cloud top height.

**Figure 2:**
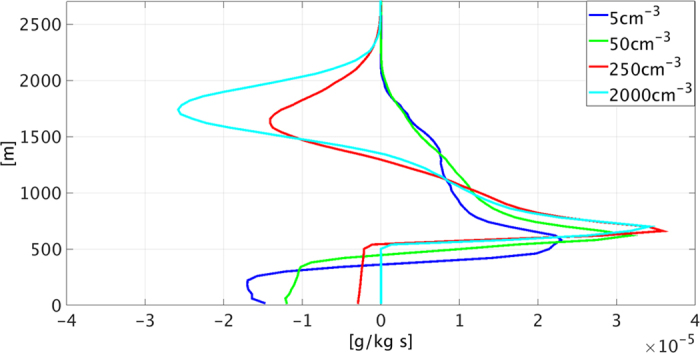
Domain’s mean condensation-less-evaporation tendencies for four different aerosol loading levels (5 cm^−3^ – blue, 50 cm^−3^ – green, 250 cm^−3^ – red, and 2000 cm^−3^ – cyan).

The evolution of the domain’s mean temperature (T) and water vapor mixing ratio (q_v_) profiles is presented in Fig. 1 for four different simulations (out of the eight conducted) with aerosol concentrations of 5, 50, 250, and 2000 cm^−3^. Hereafter, we refer to the 5 and 50 cm^−3^ simulations as clean, and the 250 and 2000 cm^−3^ simulations as polluted. The focus is on the relative differences between clean and polluted conditions, rather than on the actual magnitude which, in addition to the clouds’ feedbacks, is also affected by other factors, such as surface fluxes and large-scale forcing (LSF). The mean vertical profiles of the condensation-less-evaporation tendencies are shown in Fig. 2.

The Hovmöller diagrams (Fig. 1) clearly show that the clouds’ effects on the T and q_v_ profiles in the clean simulations are opposite in trend to those in the polluted simulations. Moreover, vertical profiles of condensation-less-evaporation tendencies (Fig. 2) reveal significant differences in the magnitude (and in some places even the sign) between the clean and polluted cases. We discuss these differences layer by layer.

### Below the cloud base (initially located at <~ 500 m)

The clean simulations (aerosol concentrations of 5 and 50 cm^−3^) exhibit a decrease in T and increase in q_v_ with time, whereas in the polluted simulations (250 and 2000 cm^−3^), there is a slight increase in T and a decrease in q_v_. These differences are mostly linked to precipitation processes^48^,^50^. In the clean simulations, rain evaporation (SI, Fig. S1) below the cloud base^28^ leads to cooling and moistening. Evaporation amounts are proportional to the rain yield, raindrop size, and the differences between saturation and the environmental relative humidity (RH). Smaller raindrops have larger resident times before reaching the surface and for a given rate of precipitation, will have a larger total surface area for evaporation. In a polluted environment the rain formation is delayed and hence starts in higher altitudes^51^ (see also Fig. S4, SI). Then, the raindrops fall along a longer path in the cloud, encountering higher droplet concentration and hence grow larger compared to raindrops in clean environments. As a result, the mean raindrop radius below the cloud base increases with the aerosol loading (up to the pollution level that shuts-off the rain – SI, Fig. S5)^34^,^51^,^52^,^53^,^54^. Hence, in the cleanest simulation, for which the raindrop radius is the smallest, the evaporative cooling below cloud base is the most significant (see Fig. 2), even though it is not the most precipitating one.

In the polluted simulations (with little or no precipitation, and hence no evaporative cooling in this layer), the sub-cloud layer warms (due to surface fluxes from below) and dries due to upward advection of humidity not fully compensated by subsidence of upper drier air (SI, Fig. S6)^35^.

### Within the cloudy layer (initially located between ~550 and 1550 m)

For the clean simulations, as the condensation-less-evaporation tendency is positive (i.e. net condensation, Fig. 2), T generally increases with time^48^. Moreover, q_v_ decreases due to stabilization of the lower atmosphere, which leads to a weaker supply of water vapor from the sub-cloud layer^46^, and removal of the water by precipitation^48^. On the other hand, due to a lack of significant rain, in the polluted simulations, q_v_ in the cloudy layer is less affected and T (especially in the upper half, ~1000–1550 m) even shows a slight decrease driven by evaporation (Fig. 2).

### The upper cloudy layer and the inversion layer (initially located between ~1550 and 2000 m)

The precipitating clean simulations and the barely precipitating polluted simulations show different behaviors. For the polluted cases, T in this layer decreases and q_v_ increases over time, mainly due to evaporation (Fig. 2). The smaller droplets in the deeper polluted clouds (SI, see mean cloud tops in Fig. S1) are carried to higher levels (as will be explained in details below) evaporate readily and therefore moisten and cool the upper cloudy layer. The overall result is deepening of the cloudy layer with time^35^,^48^. This trend is shown by the increase in maximum cloud top height altitude (marked in black lines in Fig. 1 and SI, Fig. S1).

In contrast, for the clean simulations, there is only a narrow layer of moderate cooling at the top of the inversion layer (driven by the LSF that is described further on). Figure 2 shows that in the clean simulations, there is no net evaporation in the upper part of the cloudy layer and the entire cloudy layer experiences net condensation and warming.

To summarize: in the clean simulations (5 and 50 cm^−3^), the condensed water sediments as rain and partially evaporates below the cloud base, causing cooling and moistening (and hence an increase in the sub-cloud layer’s RH–SI, Fig. S3). Under polluted conditions (250 and 2000 cm^−3^), evaporation in the upper cloudy and inversion layers is significant whereas in the sub-cloud layer, it is not. This leads to warming of the lower part of the cloudy layer and cooling and moistening of the upper part of the cloudy and inversion layers (and hence an increase in inversion layer RH). We note that the overall change in the vertical temperature and humidity profiles in time includes advection of heat and moisture between the layers in addition to the contribution of condensation and evaporation. However, our results show that the impact of condensation and evaporation controls much of the aerosol effect (see Fig. S6, SI).

The net condensation in the cloudy layer and net evaporation in the sub-cloud layer of the clean simulations result in a decrease in thermodynamic instability over time. In the polluted runs, the net condensation in the lower cloudy layer and net evaporation in the upper cloudy and inversion layers are translated into increased thermodynamic instability and convection intensity.

**Figure 3:**
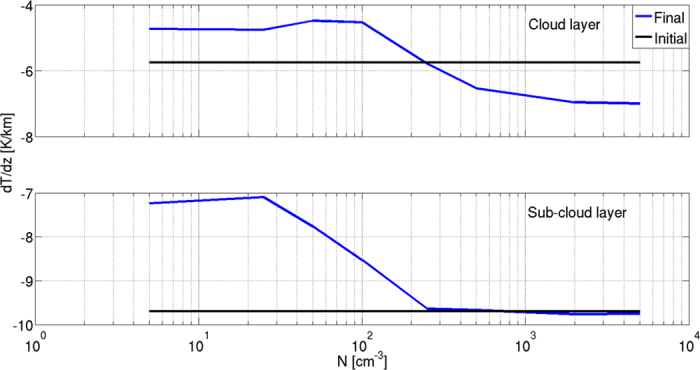
Temperature lapse rate as a function of aerosol loading (N) in the sub-cloud layer (Γ_sub,_ lower panel) and cloudy layer (Γ_c,_ upper panel). The final (after 16 h of simulation–blue) and initial (black) temperature lapse rates are presented.

Changes in thermodynamic instability can be captured in a concise way as changes in the atmospheric thermal lapse rate (Γ). Figure 3 presents the initial and final (after 16 h of simulation) Γ in the sub-cloud (Γ_sub_, for H ≤ 500 m) and cloudy (Γ_c_, 540 < H < 1500 m) layers. The initial Γ_sub_ in all simulations is close to the dry adiabatic lapse rate (−9.7 °/km), while the initial Γ_c_ characterizes a conditionally unstable atmosphere (−5.7 °/km). Under clean conditions (<100 cm^−3^), both Γ_sub_ and Γ_c_ decrease with time. For polluted conditions (>250 cm^−3^), Γ_sub_ remains almost constant and Γ_c_ is significantly larger than the initial Γ_c_, indicating an increase in the instability of the cloudy layer (~7 °/km). Figure 3 demonstrates the transition from clean clouds consuming the instability to polluted clouds enhancing it. For this specific initial profile, the transition occurs at aerosol concentrations between 100 and 250 cm^−3^. Such concentrations also mark the shift between cloud suppression and cloud deepening with time (SI, Fig. S1 for the clouds’ maximum top).

**Figure 4:**
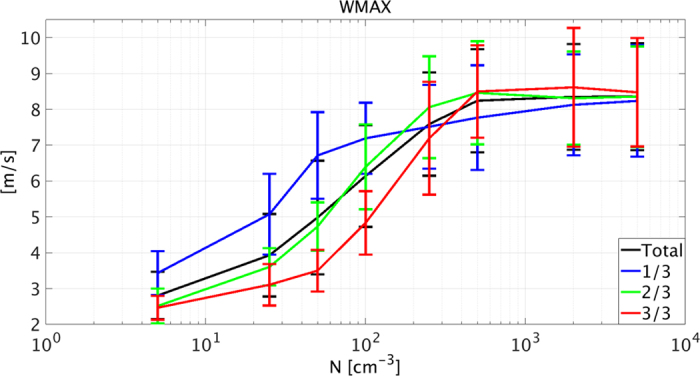
Mean over time of cloud field domain maximum vertical velocity (WMAX) as a function of the aerosol loading used in the simulation (N). Mean calculated for the last 14 h out of the 16 h of the simulation (black), and for each third of the simulation period (blue, green and red for the first, second and third periods, respectively). Error bars represent standard deviation.

Along the same lines, the maximum vertical velocity (WMAX) reflects the impact of changes in thermodynamic instability on convection intensity. The black curve in Fig. 4 presents the mean value of the entire simulation (excluding the first 2 h of spin up time), while the blue, green and red curves represent the first, second and third periods of the simulation (4.6 h each). Under clean conditions (<100 cm^−3^), WMAX decreases with time (blue, green, and red curves) whereas under polluted conditions (>250 cm^−3^) it increases, indicating an increase in thermodynamic instability.

### The velocity of the cloud’s center of gravity (COG) as a measure of instability change with time

The effective terminal velocity of a given volume in the cloud, containing air and drops (η-calculated as the mean weighted by mass droplet terminal velocity^7^) is a measure of the liquid water COG terminal velocity (the COG altitude is the average height of the cloud weighted by the mass^55^,^56^). Lower |η| values imply that the droplets move more closely with the surrounding air velocity. High aerosol loading shifts the droplet size distribution to smaller values and delays the onset of significant collection processes. Both effects imply significantly lower |η| values for longer times and therefore, the liquid water mass can be pushed higher in the atmosphere (also due to the stronger updrafts, Fig. 4). The hydrometeors’ absolute velocity, defined as the sum of the air’s mean vertical velocity (W, weighted by the water mass) and the drops effective terminal velocity (η, always negative), describes the overall vertical movement of the liquid water COG (V_COG_). Positive (negative) values of *V*_*COG*_ imply upward (downward) net vertical movement of the liquid water.

**Figure 5:**
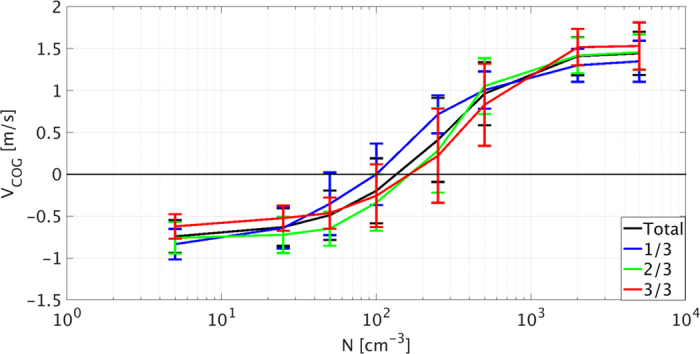
The cloud fields’ mean value of COG vertical velocity (V_*COG*_). This was calculated for the last 14 h out of the 16 h of the simulation (black) and for consecutive thirds of the simulation time (blue, green, and red for the first, second, and third parts, respectively). The standard deviation is presented as well.

Figure 5 shows the domain’s *V*_*COG*_ evolution with time for all aerosol levels. The black curve represents the mean value of the entire simulation time (excluding the first 2 h of spin up time) while the blue, green and red curves represent the first, second and third periods of the simulation (4.6 h each). This figure clearly shows that the *V*_*COG*_ values increase with increasing aerosol loading and shift from negative to positive for aerosol concentrations higher than 250 cm^−3^. Figure 2 showed net condensation in the cloudy layer and net evaporation in the sub-cloud layer for clean conditions, indicating net transport of the liquid water from the cloudy layer to the sub-cloud layer (downward gradient of the condensation-less-evaporation profile). The water that condenses in the cloudy layer sediments down to the sub-cloud layer where it partially evaporates. On the other hand, for the polluted cases, *V*_*COG*_ is positive, indicating that the net liquid water movement is upward. The water that is being condensed in the lower part of the cloudy layer is transported upward and evaporates in the upper cloudy and inversion layers (Fig. 2).

The differences in the values of *V*_*COG*_ between the clean simulations, with aerosol loading below 100 cm^−3^ (negative values) and the polluted simulations, with aerosol concentration above 250 cm^−3^ (positive values) can explain the observed shift between increased and decreased instability in Fig. 3. We also note that the aerosol level of the crossing point from negative to positive values of *V*_*COG*_ increases with time (blue, green and red curves) from about 100 cm^−3^ to about 200 cm^−3^. This implies that the cloud’s deepening mechanism ultimately serves to suppress itself, as has been previously shown^35^. Clouds that hardly precipitate at the initial stages of the simulation will precipitate at later stages (due to the deepening effect^35^) and will therefore eventually consume the instability, shifting the overall COG movement from *V*_*COG*_ > 0 to *V*_*COG*_ < 0. Given enough time, the polluted clouds will become sufficiently deep for precipitation to form and the increase in instability will cease^35^. As was recently shown, the time required for sufficient deepening increases with aerosol loading: a larger increase in the instability during a longer time, up to the initiation of precipitation^35^. We conducted one longer simulation (32 h for the 500 cm^−3^ case) and it shows that the clouds’ deepening trend becomes more gradual with time.

We note that the LSF setup in the model (including the effects of vertical and horizontal advection of heat and moisture and radiative cooling) also affects the evolution of the thermodynamic conditions in the field^48^,^57^,^58^,^59^. The magnitude and even the sign of the changes in temperature and humidity profiles with time might be different for different LSF setups. Nevertheless, we show that the net aerosol effect on the profiles show the same trends. To demonstrate this point, four additional simulations were conducted: with no LSF and with two times the standard BOMEX LSF, each case with aerosol concentrations of 50 cm^−3^ (clean) and 2000 cm^−3^ (polluted) (see Table S1 for details and Fig. S2 in the SI). Consistent with the results above, under clean precipitating conditions, the cloudy layer becomes warmer while the sub-cloud layer becomes colder compared to the polluted non-precipitating simulations for all LSF setups. The inversion layer in the polluted simulations becomes colder compared to the clean simulations, regardless of the magnitude of the LSF. Another source of uncertainty is the domain size. For examining it three additional simulations were conducted with horizontal domain size of 51.2 × 51.2 km^2^, for half of the regular simulation time (8 h). The main conclusions of this work were found to be valid in those large domain simulations as well (see the SI for details, Figs S7–8).

In this work, the effects of clouds on their environment were shown by tracing the evolution of the temperature and humidity profiles (Fig. 1), and specifically of the temperature lapse rate (Fig. 3). The clouds’ effects on the environmental conditions were found to be regulated by the aerosol concentration, via its control on cloud processes such as precipitation production, condensation and evaporation (Fig. 2), and droplet mobility (Fig. 5).

Clean conditions promote the formation of precipitation and hence stabilize the environment, whereas polluted conditions suppress precipitation; the smaller droplets therefore evaporate higher in the atmosphere, destabilizing the environment and invigorating the convection (Fig. 4). The magnitude and sign of these effects can be measured using the absolute velocity of the cloud field’s liquid water COG (summation of the air velocity and the droplet terminal velocities, all weighted by liquid mass, i.e. *V*_*COG*_), where positive (negative) values dictate destabilization (stabilization).

Clouds alter the thermodynamic properties of the field in which they form. Their interaction with the environmental thermodynamic properties strongly depends on the outcomes of microphysical processes, and therefore on aerosol loading. As clouds regulate the incoming planetary solar radiation, such effects on cloud field trends could have strong implications for overall cloud forcing.

### Methodology

#### Model and setup

The SAM (System for Atmospheric Modeling) LES model version 6.10.3^60^ (for details see webpage: http://rossby.msrc.sunysb.edu/~marat/SAM.html) was used to simulate the BOMEX case study, which is one of the best known benchmarks for trade-cumulus clouds^61^,^62^. BOMEX simulates an idealized trade-wind cumulus cloud field based on detailed measurements made near Barbados in June 1969. This case was initialized using the standard setup specified in *Siebesma et al*.^62^. The horizontal resolution was set to 100 m and the vertical resolution to 40 m. The domain size was 12.8 × 12.8 × 4.0 km^3^ and the time step was 1 s. The model ran for 16 h and the statistical analysis included all but the first 2 h (total of 14 h). A bin microphysical scheme^63^ was used. The scheme solves warm microphysical processes, including droplet nucleation, diffusional growth, collision–coalescence, sedimentation and breakup.

The aerosol size distribution was based on marine size distribution^64^. Eight different simulations were conducted with aerosol concentrations of 5, 25, 50, 100, 250, 500, 2000 and 5000 cm^−3 ^5. To avoid giant CCN effects, aerosols with radius >2 μm were cut from the distribution^17^,^65^,^66^. The smallest aerosol bin used was 5 nm.

For isolating the aerosol effect on the thermodynamic conditions, the radiative effects (as included in the LSF) as well as the surface fluxes were prescribed in all simulations.

Due to computational limitations, the domain size was restricted to 12.8 km. We note that such a scale is limited in capturing large-scale organization^16^. For examining the sensitivity of the main conclusions to the domain size three additional large-domain simulations were conducted (see the SI for details).

## Additional Information

**How to cite this article**: Dagan, G. *et al*. Aerosol effect on the evolution of the thermodynamic properties of warm convective cloud fields. *Sci. Rep.* **6**, 38769; doi: 10.1038/srep38769 (2016).

**Publisher's note:** Springer Nature remains neutral with regard to jurisdictional claims in published maps and institutional affiliations.

## Supplementary Material

Supplementary Information
