# Supplementary material for: Aerosol effect on the evolution of the thermodynamic properties of warm convective cloud fields
Source: Sci Rep. 2016 Dec 8;6:38769. doi: 10.1038/srep38769 (PMC5144091; doi:10.1038/srep38769)
Supplement: Supplementary Information [file srep38769-s1.pdf]

Supporting Information for

Aerosol effect on the evolution of the thermodynamic properties of warm convective cloud fields

Guy Dagan, Ilan Koren*, Orit Altaratz and Reuven H. Heiblum

Department of Earth and Planetary Sciences, The Weizmann Institute of Science, Rehovot 76100, Israel

*Corresponding author. E-mail: ilan.koren@weizmann.ac.il

Content of this file

Text:

S.1 Clouds' mean properties.

S.2 Large-scale forcing effects on the thermodynamic conditions.

S.3 Changes in mean RH profile with time.

S.4 Information regarding the vertical profiles of hydrometeors' amount and size.

S.5 Contribution of vertical advection versus condensation/evaporation and LSF to the changes in temperature and humidity profiles.

S.6 Sensitivity of the results to domain size.

Figures S1-S8.

S.1 Clouds' mean properties

Figure S1 presents clouds' mean properties (rain rate and maximum cloud top height) in eight different simulations (5, 25, 50, 100, 250, 500, 2000 and 5000 cm^{-3}) as a function of the aerosol loading used in the simulation. The black curves show global averages (over 14 h) as a function of aerosol concentration, while the blue, green and red curves represent the mean values over the first, second and third periods of the

simulation, respectively (each period lasting 4 h and 40 min: blue 0200–0640 h, green 0640–1120 h, and red 1120–1600 h).

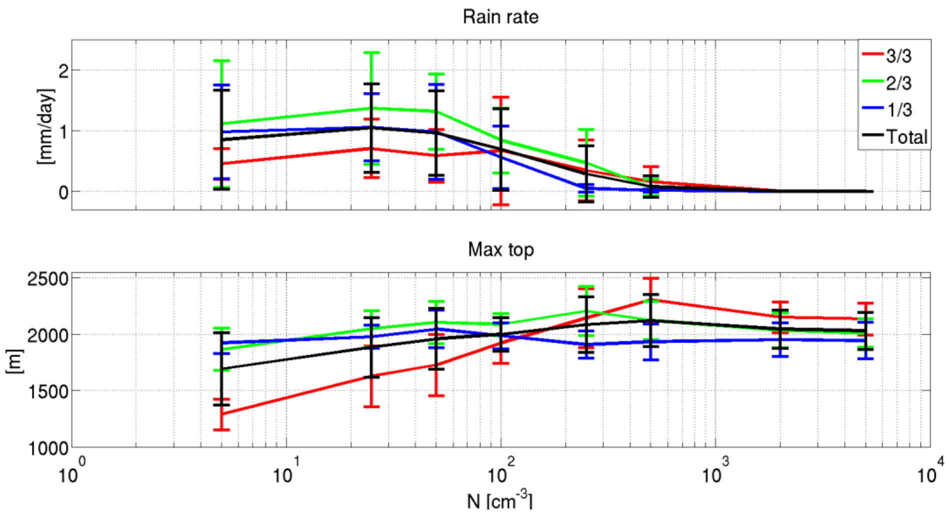

Figure S1. **Mean over time of cloud field domain properties as a function of the aerosol loading used in the simulation (N). Upper panel – mean domain surface rain rate, and lower panel – maximum domain cloud top height. The mean of each of these properties was calculated for the last 14 h out of the 16 h of the simulation (black) and for each third of the simulation period (blue, green and red for the first, second and third periods, respectively). Error bars represent standard deviation.**

S.2 Large-scale forcing effects on the thermodynamic conditions

Among other things examined in the paper (like cloud feedbacks) the LSF affects the evolution of the thermodynamic conditions in the field as well[1-4]. In the BOMEX case study, the LSF includes the effects of subsidence (negative vertical velocity) and advection that act to cool and dry the profile (see Table S1). The magnitude and even the sign of the temporal changes in temperature might be different for different LSF setups, but the overall aerosol effect on the profiles is expected to be the same (for a certain range of LSF setups that support clouds formation). To demonstrate this point, additional simulations with no LSF and with two times the standard BOMEX LSF were conducted. Each LSF setup was simulated with two different aerosol loading levels: 50 cm^{-3} (clean) and 2000 cm^{-3} (polluted). The changes in the virtual potential temperature profile with time are shown in Fig. S2. Under clean precipitating

conditions, the cloudy layer (~550–1550 m) becomes warmer with time, while the sub-cloudy layer becomes colder compared to the polluted non-precipitating simulations. The inversion layer (~1550–2000 m), in the polluted simulation becomes colder compared to the clean simulation regardless of the magnitude of the LSF.

Table S1. The large-scale forcing (LSF) standard setup used in the BOMEX case study simulations. The LSF includes temperature, water vapor mixing ratio and vertical velocity tendencies as a function of height.

Z [m]	Temperature tendency by LSF [K/s]	Water vapor mixing ratio tendency by LSF [kg/kg s]	Vertical velocity tendency by LSF [m/s]
0	$-2.315 \cdot 10^{-5}$	$-1.2 \cdot 10^{-8}$	0
300	$-2.315 \cdot 10^{-5}$	$-1.2 \cdot 10^{-8}$	-0.0013
500	$-2.315 \cdot 10^{-5}$	0	-0.0021
1500	$-2.315 \cdot 10^{-5}$	0	-0.065
2100	$-1.389 \cdot 10^{-5}$	0	0
2500	0	0	0

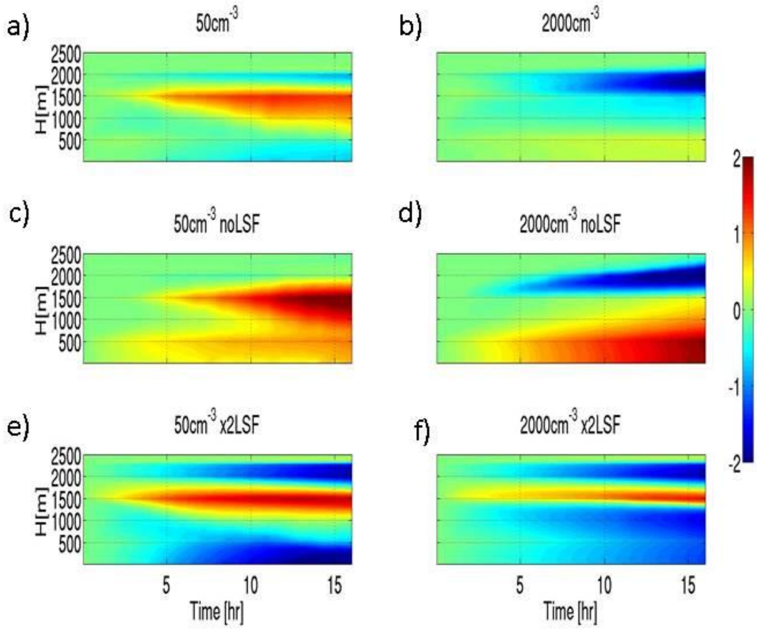

Figure S2. Changes in the domain mean virtual potential temperature [K] profile as a function of time. Each subplot represents one simulation conducted under different aerosol loading conditions (50 and 2000 cm^{-3}) and different large-scale forcing (LSF) conditions: the standard BOMEX LSF (panels a and b), no LSF (noLSF – panels c and d), and two times the standard BOMEX LSF (x2LSF – panels e and f).

S.3 Changes in mean RH profile with time

The changes in the environmental RH with time as a function of height are presented in Fig. S3 for four simulations (5, 50, 250 and 2000 cm^{-3}). The temporal change of the RH is a function of the changes in both temperature and water vapor mixing ratio (both presented in Fig. 1 in the main text).

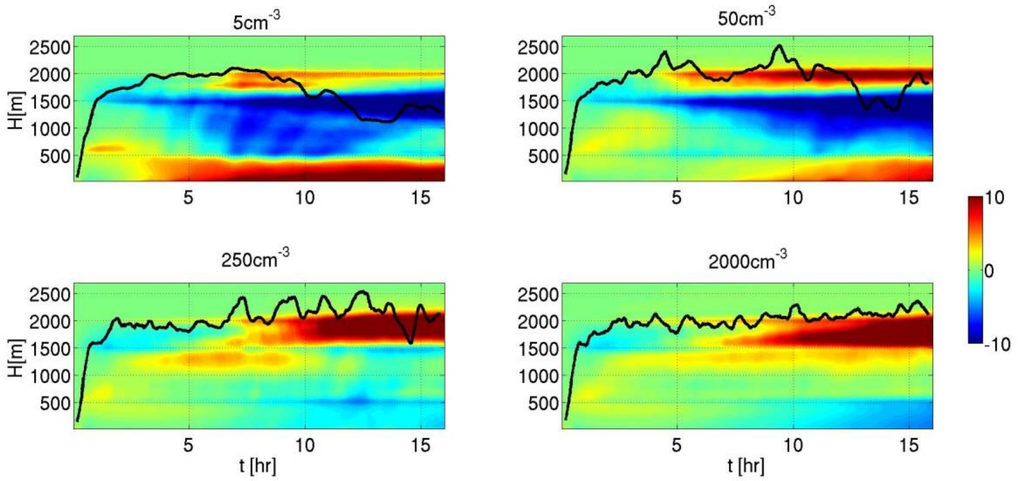

Figure S3. Changes in the domain mean relative humidity profile as a function of time. Each subplot represents one simulation conducted under different aerosol loading conditions (5, 50, 250 and 2000 cm^{-3}). Black lines present the 10 minutes running average of the maximum cloud top height.

The black lines represent the maximum cloud top height. To reduce the noise we present 10 minutes running average.

S.4 Information regarding the vertical profiles of hydrometeors' amount and size

Figure S4 presents the mean vertical profile of rain-drops mixing ratio (defined according to a threshold of 40 μm radius) for four simulations. It demonstrates the increase in raindrops maximal height together with the increase in aerosol loading.

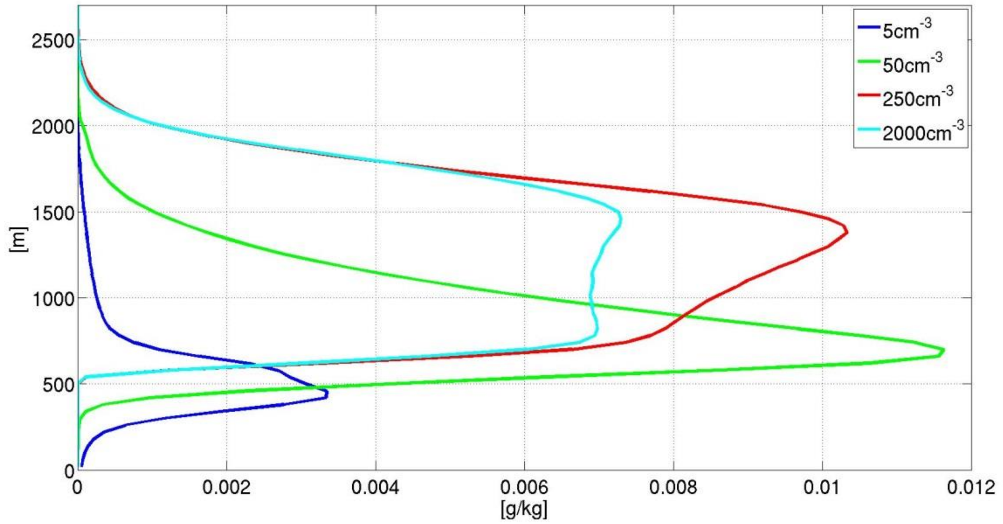

Figure S4. Mean (over time and domain) vertical rain drop mixing ratio profile for four simulations (with aerosol loading levels of 5 cm^{-3} – blue, 50 cm^{-3} – green, 250 cm^{-3} – red, and 2000 cm^{-3} – cyan). Rain drops are defined according to a threshold of 40 µm radius.

Figure S5 presents vertical profiles of the mean drop radius for four simulations differ by the aerosol loading. In the cloudy and inversion layers (H>550m) the mean drop radius decreases with increasing aerosol loading. Below cloud base it reverses and the mean raindrop radius increases together with the aerosol loading. This increase in the rain-drop size act to reduce the rain evaporation in those polluted simulations.

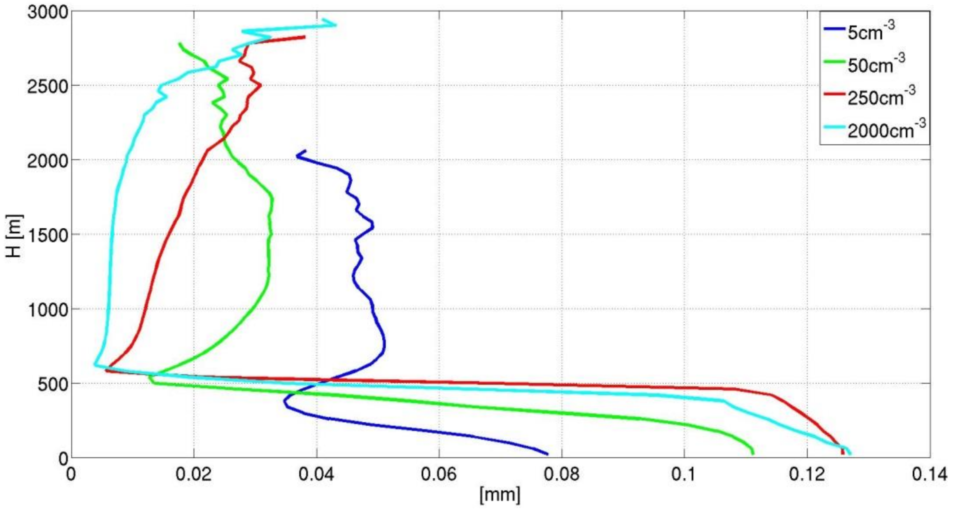

Figure S5. Vertical profiles of the mean drops radius (including both cloud and raindrops) for four simulations (with aerosol loading levels of 5 cm^{-3} – blue, 50 cm^{-3} – green, 250 cm^{-3} – red, and 2000 cm^{-3} – cyan).

S.5 Contribution of vertical advection versus condensation/evaporation and LSF to the changes in temperature and humidity profiles

The figure below (Fig. S6) presents the net heat (upper panel) and humidity (lower panel) change in each height driven by vertical advection (Figs. S6a and S6d), condensation/evaporation tendencies (Figs. S6b and S6e - which are presented in Fig. 2 of the main text), and LSF (Figs. S6c and S6f). Below cloud base the vertical heat advection results in warming of the sub-cloud layer in all simulations. The heat source is the surface fluxes. On the other hand, the evaporation process results in cooling of the sub-cloud layer only in the clean simulation. Those two effects, together with the LSF prescribed by the model set-up (drives the same cooling in all simulations) result in cooling (warming) of the sub-cloud layer in the clean (polluted) simulation. We note that although all three terms are non-negligible, the main difference in the mean heat and humidity profiles between clean and polluted simulations is contributed by the condensation/evaporation tendency. It drives net cooling of the sub cloud layer under clean conditions (Fig. 1 in the main text) which does not happen in the polluted conditions, and strong cooling of the upper cloudy and inversion layer under polluted conditions.

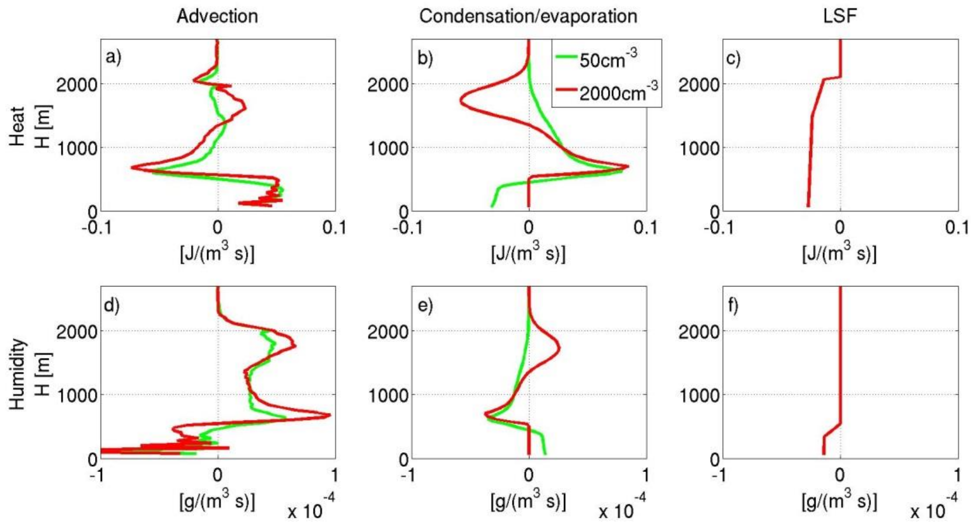

Figure S6. Mean profiles (over domain and time) of net heat (a-c) [J/(m^3 s)] and humidity (d-f) [g/(m^3 s)] change (per height) driven by vertical advection (a and d), condensation and evaporation (b and e), and large scale forcing (c and f), for two simulations (50, and 2000 cm^{-3}).

S.6 Sensitivity of the results to domain size

The domain size was shown to be a key player in determining warm cloud field properties[5]. For examining the sensitivity of our conclusions to the domain size we have conducted three additional simulations with larger horizontal domain size of 51.2x51.2 km^2 with the same vertical grid (40m resolution) and horizontal resolution (100m). Due to computational limitations only three (5, 50 and 500 cm^{-3}) large simulations for half of the regular simulation time (8h) were conducted. The results (presented in Figs. S7-8) demonstrate that the general trends of an increase in the instability under polluted conditions and consumption of the instability under clean conditions are valid for the larger domain simulations as well.

Temp change:

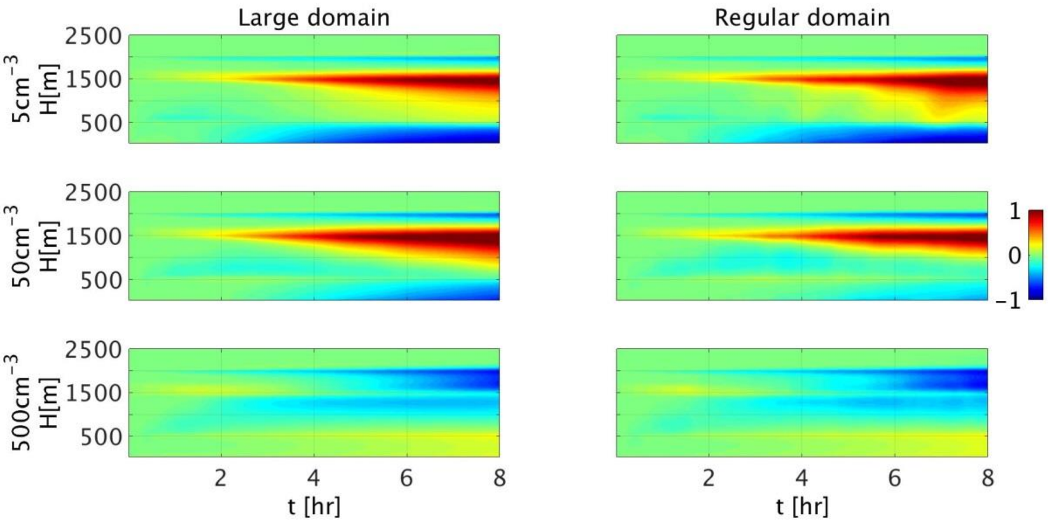

Figure S7. Temporal changes compared to the initial profiles of mean temperature [K]. Each row shows the temporal evolution of the differences for a given simulation (with aerosol concentration of 5, 50 and 500 cm^{-3}). Left column presents the results of large domain simulations (51.2x51.2km^2) while the right column presents the regular domain simulations (12.8x12.8km^2).

Qv change:

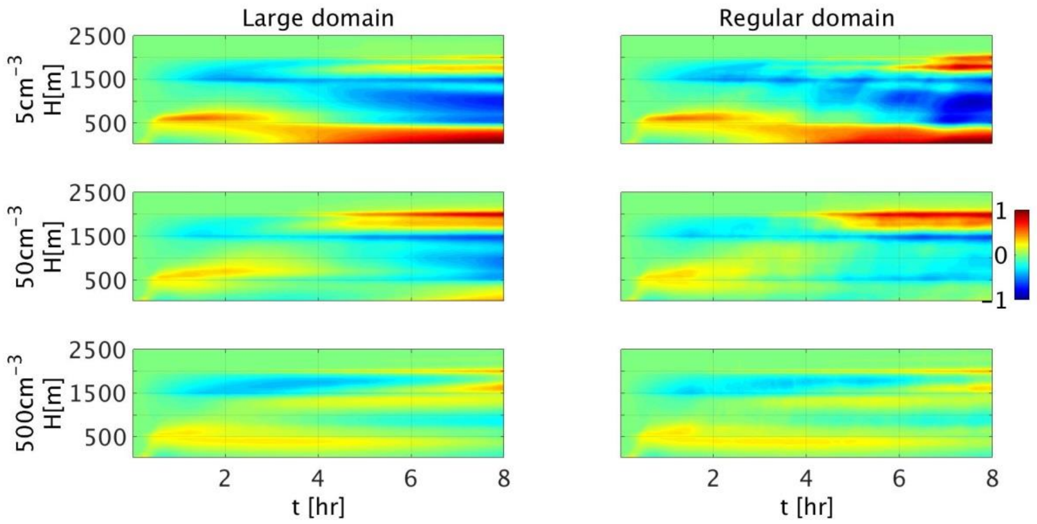

Figure S8. Temporal changes compared to the initial profiles of mean water vapor mixing ratio [g/kg]. Each row shows the temporal evolution of the differences for a given simulation (with aerosol concentration of 5, 50 and 500 cm^{-3}). Left column presents the results of large domain simulations (51.2x51.2km^2) while the right column presents the regular domain simulations (12.8x12.8km^2).

<u>References</u>

1. Malkus JS (1958) On the structure of the trade wind moist layer.
2. Albrecht BA (1993) Effects of precipitation on the thermodynamic structure of the trade wind boundary layer. *Journal of Geophysical Research: Atmospheres (1984–2012)* 98(D4):7327-7337.
3. Albrecht BA, Betts AK, Schubert WH, & Cox SK (1979) Model of the thermodynamic structure of the trade-wind boundary layer: Part I. Theoretical formulation and sensitivity tests. *Journal of the Atmospheric Sciences* 36(1):73-89.
4. Soong S-T & Ogura Y (1980) Response of tradewind cumuli to large-scale processes. *Journal of the Atmospheric Sciences* 37(9):2035-2050.
5. Seifert, A. & Heus, T. Large-eddy simulation of organized precipitating trade wind cumulus clouds. *Atmospheric Chemistry and Physics* **13**, 5631-5645 (2013)1.7
